# Prevalence of sleep disturbances and long-term reduced health-related quality of life after critical care: a prospective multicenter cohort study

**DOI:** 10.1186/cc6973

**Published:** 2008-08-01

**Authors:** Lotti Orwelius, Anders Nordlund, Peter Nordlund, Ulla Edéll-Gustafsson, Folke Sjöberg

**Affiliations:** 1 Department of Intensive Care, Division of Perioperative Medicine, Linköping University/Linköping University Hospital, Garnisonsvägen, 581 85, Linköping, Sweden; 2 Department of Medicine and Care, Nursing Science, Linköping University/Linköping University Hospital, Garnisonsvägen, 581 85 Linköping, Sweden; 3 Department of Hand and Plastic Surgery, Division of Clinical and Experimental Medicine, Faculty of Health Sciences, Linköping University/Linköping University Hospital, Garnisonsvägen, 581 85; 4 TFS Trial Form Support AB, 222 28 Lund, Sweden; 5 Department of Anaesthesia and Intensive Care, Intensiv Care Unit, Ryhov Hospital, 551 85 Jönköping, Sweden

## Abstract

**Introduction:**

The aim of the present prospective multicenter cohort study was to examine the prevalence of sleep disturbance and its relation to the patient's reported health-related quality of life after intensive care. We also assessed the possible underlying causes of sleep disturbance, including factors related to the critical illness.

**Methods:**

Between August 2000 and November 2003 we included 1,625 consecutive patients older than 17 years of age admitted for more than 24 hours to combined medical and surgical intensive care units (ICUs) at three hospitals in Sweden. Conventional intensive care variables were prospectively recorded in the unit database. Six months and 12 months after discharge from hospital, sleep disturbances and the health-related quality of life were evaluated using the Basic Nordic Sleep Questionnaire and the Medical Outcomes Study 36-item Short-form Health Survey, respectively. As a nonvalidated single-item assessment, the quality of sleep prior to the ICU period was measured. As a reference group, a random sample (n = 10,000) of the main intake area of the hospitals was used.

**Results:**

The prevalence of self-reported quality of sleep did not change from the pre-ICU period to the post-ICU period. Intensive care patients reported significantly more sleep disturbances than the reference group (*P *< 0.01). At both 6 and 12 months, the main factor that affected sleep in the former hospitalised patients with an ICU stay was concurrent disease. No effects were related to the ICU period, such as the Acute Physiology and Chronic Health Evaluation score, the length of stay or the treatment diagnosis. There were minor correlations between the rate and extent of sleep disturbance and the health-related quality of life.

**Conclusion:**

There is little change in the long-term quality of sleep patterns among hospitalised patients with an ICU stay. This applies both to the comparison before and after critical care as well as between 6 and 12 months after the ICU stay. Furthermore, sleep disturbances for this group are common. Concurrent disease was found to be most important as an underlying cause, which emphasises that it is essential to include assessment of concurrent disease in sleep-related research in this group of patients.

## Introduction

Intensive care affects the patients in many ways, and also influences the outcome after discharge [1,2]. After a period in intensive care, patients have reported poorer health-related quality of life (HRQoL) compared with a reference group [3]. Furthermore, in a previous study we found that this poorer HRQoL is mostly the result of the high prevalence of concurrent disease among the patients rather than due to factors related to intensive care [4]

Sleep is important for overall wellbeing [5]. In the short term, we know that many patients, irrespective of their diagnosis, have disturbed sleep during their time in the intensive care unit (ICU) and up to 1 week afterwards [6-9]. Former ICU patients may have more short-term sleep disturbances caused by both the period of critical care and the high prevalence of concurrent diseases [4]. Sleep-related problems may persist long after the patients have left the ICU. Because of the paucity of studies, however, the prevalence and extent of sleep disturbances that remain long term (>3 months) after intensive care are unknown. A partly unanswered question is also the effect of sleep disturbances on HRQoL of former ICU patients. There is a difficulty in assessing sleep disturbances, as sleep varies with sex [10,11] and with age [11]. Sleep disturbance is also affected by concurrent diseases [12], so a reliable reference group is essential to be able to evaluate the prevalence of sleep disturbances properly.

The aim of the present study was to investigate the long-term (6-month and 12-month) sleep pattern after critical illness. We also wanted to examine specifically the relation between sleep disturbances and HRQoL. Furthermore, we wanted to know whether concurrent disease and factors related to intensive care (Acute Physiology and Chronic Health Evaluation (APACHE) II, length of stay, and admission diagnosis) affected the long-term sleep patterns in the ICU group.

We hypothesised that hospitalised patients with an ICU stay have an affected sleep long after the intensive care period has ended, but we suspected that it is the result of concurrent disease rather than of ICU-related factors.

## Materials and methods

### Design

The present prospective, longitudinal study was carried out between August 2000 and November 2003 in three general ICUs in Sweden: one university hospital, and two general hospitals. The ICU at the university hospital has eight beds, and 500 to 750 patients are admitted annually. Postoperative patients, those after open-heart surgery and neurosurgery, those with primary coronary disease, neonates, and burned patients are treated in other specialised units, and were not included in the present study. The two general hospitals both have six-bed ICUs, and 500 to 700 patients are admitted annually to each. The units are the only ICUs at the hospitals except for the care of neonates. Over 90% of the admissions to these three ICUs are emergencies, and the primary admission diagnoses are most commonly multiple trauma, sepsis, and disturbances in the respiratory or circulatory systems, or both. All adults (18 years old and over) who were consecutively admitted and who remained in the ICU for more than 24 hours, and who were alive 6 months after discharge from hospital, were included. Patients who were readmitted were included only for their first admission. This database has previously been used and will be used in several outcome studies in critical care [4].

The clinical databases in each hospital were used to extract data on age, sex, reason for admission to and length of stay in the ICU, APACHE II score [13], length of stay in hospital, and outcome. Admissions were categorised into diagnostic groups: multiple trauma, sepsis, respiratory, gastrointestinal, cardiovascular, and other.

The design of the study was approved by the Committee for Ethical Research at the University of Health in Linköping. Eligible patients consented to participate in the study.

### Participants

**Figure 1:**
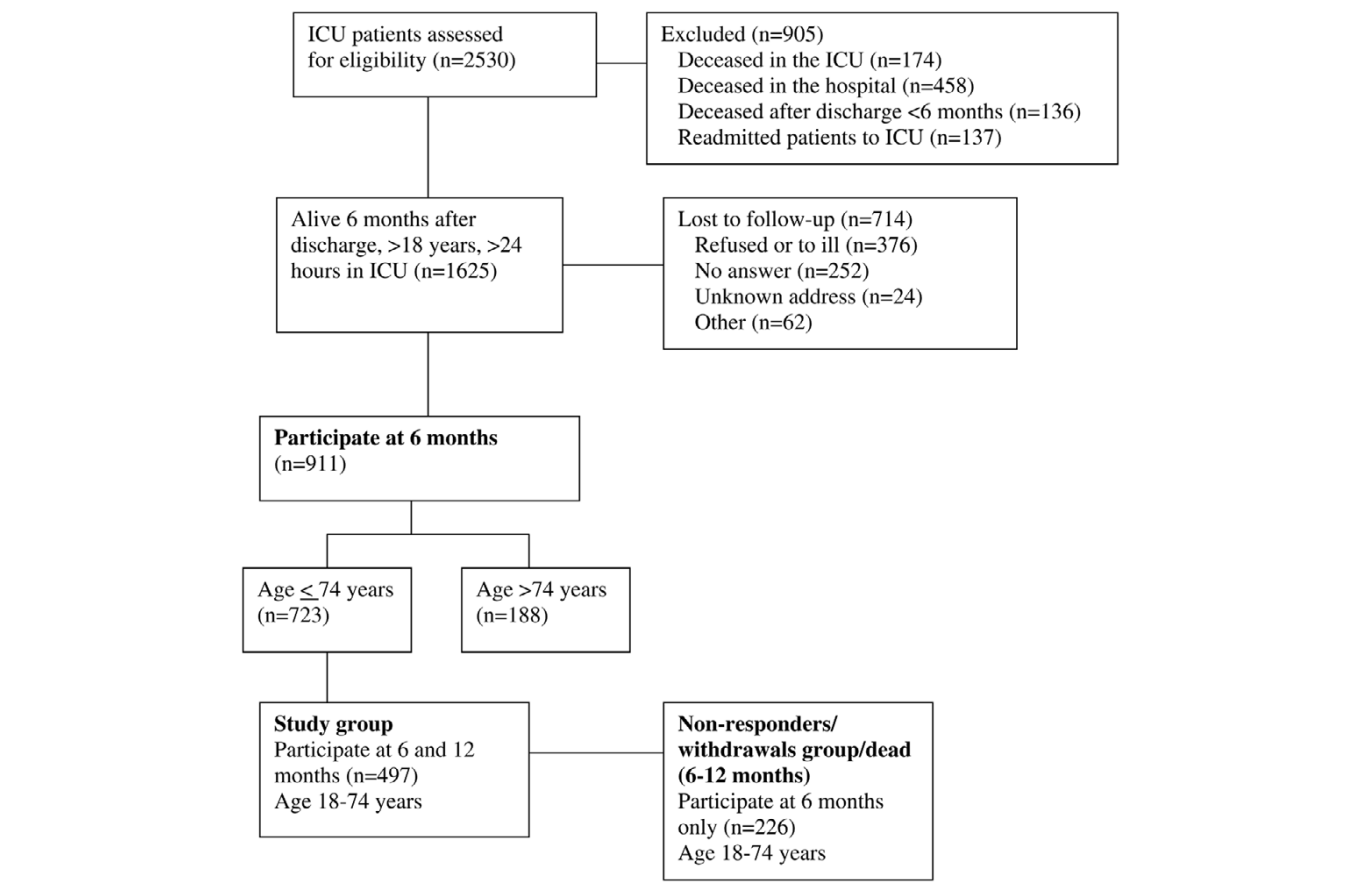
Algorithm of patients who were and were not included in the sleep disturbance study. All patients that responded at 6 months were used in baseline comparisons, whereas patients that responded both at 6 and 12 months and were younger than 75 years old were used in comparison with the reference group. ICU, intensive care unit.

A total of 1,625 patients met the inclusion criteria. Of these, 911 patients answered the questionnaire at 6 months and are used in the baseline comparisons. In order to achieve comparability with the reference group, 188 patients were excluded because they were older then 74 years of age, the upper age limit for the sample from the reference group. Of the patients between 18 and 74 years old, 723 responded to the first inquiry at 6 months and 497 also responded at 12 months, and they then became the study group and are used in the comparisons with the reference group (Figure 1).

For the reference group, data from a public health survey of the county of Östergötland (the area in which the university hospital and one of the general hospitals is situated, adjacent to the county where the second general hospital is located) were used for comparison of sleep disturbances, concurrent disease and HRQoL. Questionnaires were initially sent out to 10,000 people. After two reminders, 6,093 (61%) had responded [14].

### Questionnaires

A set of structured questionnaires with information about the study and a request to participate were sent to the surviving patients 6 and 12 months after their discharge from hospital. The questionnaire contained questions about the patients' background data, including concurrent disease (self-reported diagnosis). The questionnaire asked 'Do you have any of the following illnesses and have had it for more than 6 months before the intensive care period with the pre-specified alternatives: cancer; diabetes; heart failure; asthma or allergy; rheumatic; gastrointestinal; blood; kidney; psychiatric; neurological disease; thyroid or any other metabolic disturbance, or other long-term illness?' (Table 1).

**Table 1:** Characteristics of patients in the study group (6 and 12 months), in the nonresponders/withdrawals at 12 months group, and in the reference group

	Study group (n = 497)	Nonresponders/withdrawals group (n = 226)	*P *value^a^	Reference group (n = 6093)	*P *value^b^
Sex (male/female)	274/223	136/90	0.23	2822/3271	**<0.0001**
Age (years)	52.4 (15.7)	52.5 (16.1)	0.97	46.4 (15.1)	**<0.0001**
Marital status			0.09		**0.006**
Married	327 (67)	130 (59)		4484 (74)	
Single	135 (28)	79 (35)		1334 (22)	
Widow/widower	27 (15)	13 (6)		274 (4)^c^	
Children at home < 19 years	116 (24)	40 (18)	0.08		
Born in Sweden	454 (92)	201 (89)	0.26	5569 (91)	0.74
Education					**<0.0001**
Compulsory school	166 (34)	84 (38)	0.35	1785 (29)	
High school/university	121 (25)	52 (23)	0.78	1371 (22)	
Employment before ICU stay			**0.04**		**<0.0001**
Employed	237 (50)	103 (48)		3589 (59)	
Retired	192 (41)	83 (39)		1145 (19)	
Student	20 (4)	6 (3)		402 (7)	
Other	21 (5)	23 (10)		957 (16)^d^	
6 months after ICU stay			0.31		
Employed	201 (44)	70 (35)			
Retired	206 (45)	101 (51)			
Student	15 (3)	9 (4)			
Other	36 (8)	20 (10)			
Sick leave before ICU stay	70 (14)	29 (13)	0.46		
Reported sick <100%	13 (3)	8 (3)			
Reported sick 100%	50 (10)	18 (3)			
6 months after ICU	121 (24)	59 (26)	0.70		
Reported sick <100%	20 (4)	8 (3)			
Reported sick 100%	94 (19)	48 (21)			
Concurrent disease^e^	342 (69)	179 (79)	**0.005**	3095 (51)	**<0.0001**
Cancer	48 (10)	32 (18)			
Diabetes	57 (11)	29 (16)			
Cardiovascular	83 (17)	36 (20)			
Gastrointestinal	50 (10)	25 (14)			
Miscellaneous	322 (65)	168 (74)			

Not all patients answered all questions. Data presented as n (%) of totals, except age as mean (standard deviation). Bold data is on significant level. ^a^Study group compared with nonresponders/withdrawals at 12 months (Fisher's exact test or Pearson chi-square test). ^b^Reference group compared with study group (Fisher's exact test or Pearson chi-square test). ^c^In public health survey, the category variable was other. ^d^Including sick leave. ^e^Patients can have more than one disease.

The questionnaire to the reference group also included, apart from questions on background characteristics, questions about health problems – including sleep and HRQoL (Medical Outcomes Study 36-item Short-form Health Survey (SF-36)).

### Instruments

#### Sleep disturbance

The questions were taken from the Swedish version of the Basic Nordic Sleep Questionnaire [15]. The instrument has been shown to be valid [15,16].

Three questions included in the Basic Nordic Sleep Questionnaire were used: 'Were there difficulties in falling asleep?' 'What was the quality of sleep like?' 'Was there a difference between the reported need for sleep and that achieved?' These questions were also used in the public health survey. To the second question above ('What was the quality of sleep like?'), yet another, single nonvalidated question [17] was added asking about the quality of sleep prior to the ICU stay. This question was only asked of the ICU group. The sleep instruments used in the study are presented in Additional file 1.

#### Health-related quality of life

The SF-36 was chosen for the evaluation of HRQoL [18,19]. The instrument is internationally well known and has often been used [20]. The SF-36 has previously been applied in intensive care [4,21,22], and has recently been recommended as one of the best-suited instrument for measuring HRQoL in trials in critical care [23].

The SF-36 has been translated into Swedish and validated in a representative sample [24]. The survey has 36 questions and generates a health profile of eight subscale scores: physical functioning, role limitations caused by physical problems, bodily pain, general health, vitality, social functioning, role limitations due to emotional problems, and mental health [18,24]. The scores on all subscales are transformed to a scale ranging from 0 (the worst score) to 100 (best score).

### Statistical analysis

Data are presented descriptively using parametric statistics (mean, 95% confidence intervals, and one-way analysis of variance) and nonparametric statistics (Pearson's chi-square test and Kruskal–Wallis test). Logistic regression analysis, adjusted for sex, age, and concurrent disease, was used to evaluate the difference between the patients and the reference groups as appropriate. Logistic regression was also used to evaluate the independent effects of sex, age, concurrent disease, APACHE II scores on admission, length of stays in ICU and in hospital, and diagnoses on admission on sleep disturbances among the patients and the relation between sleep disturbances and HRQoL.

Sleep disturbances and HRQoL among the ICU patients were compared with those reported by the sample of the general population of the county of Östergötland, who had answered an independent mail survey in 1999. There were three questions in this mail survey that overlapped with questions on sleeping problems in our study. The answers were dichotomised and compared as follows: the severity of difficulties in falling asleep at least weekly rather than less than weekly; poor quality of sleep or worse compared with good or better sleep; and time slept less than required compared with time slept equal to or more than required.

Interactions were also assessed. As eight different HRQoL measures were used (the SF-36 eight subscales), the number of comparisons involved became rather large. No adjustment for multiple comparisons was done. Findings were considered significant, however, only if there were concurrent changes in several related variables.

The Statistical Package for the Social Sciences (version 15.0; SPSS Inc., Chicago, IL, USA) was used for the statistical analyses. *P *< 0.05 was accepted as significant.

## Results

### Characteristics of patients

The characteristics of the patients in the study group, in the nonresponders/withdrawals group at 12 months, and in the reference group are presented in Table 1.

The patients in the study group (n = 497) were less out of work and were less likely to have concurrent diseases than the patients in the nonresponding/withdrawals group (n = 226). Compared with the reference group, the patients in the study group were more likely to be men, to be older, to have different marital status and education status, and to be retired. The study group patients also more often had concurrent diseases in the same comparison (69% versus 51%).

There were no significant differences between the study group and the nonresponders/withdrawals group in the APACHE score (*P *= 0.106), the length of stay in the ICU (*P *= 0.130) or the length of stay in the hospital (*P *= 0.474), or in the diagnoses recorded at admission (*P *= 0.899), the most common of which was gastrointestinal disease (data not shown).

### Sleep disturbances

In comparing the quality of sleep pattern prior to the ICU stay with that 6 months after the ICU/hospital discharge, the prevalence of self-reported quality of sleep did not change from the pre-ICU period to the post-ICU period (Table 2).

**Table 2:** Comparison of quality of sleep before the intensive care unit (ICU) period and 6 months after ICU period (n = 911)

Before ICU stay^a^	6 months after ICU stay^b^	
	
	Good	Bad
Good	459 (70)	60 (9)
Bad	56 (8)	85 (13)

Seventy-two percent of the patients answered the questions (a) Rate your overall sleep quality before the intensive care period, and (b) Rate your overall sleep quality during the last month. Data presented as number (%) of totals.

The study group had more difficulty in falling asleep, had poorer quality of sleep and slept for shorter periods than the reference group (38% versus 13%, 20% versus 12% and 61% versus 55%, respectively). Apart from difficulties falling asleep, these differences were minor after adjusting for sex, age and concurrent disease. Little or no improvement was seen over time for the ICU group in falling asleep, quality of sleep, and sleep deficit (data not shown). When we compared the previously healthy in the study group with those with concurrent diseases, difficulty in falling asleep and quality of sleep increased and decreased by almost 50%, respectively. When the study group with concurrent disease was compared with the corresponding people in the reference group, the quality of sleep and amount of sleep deficit were roughly the same (Table 3). For the hospitalised patients with an ICU stay, the clinical data did not differ for the two groups presenting sleep disturbances at 6 months and presenting no sleep disturbances at 6 months (n = 911) (Table 4).

**Table 3:** Sleep disturbances in patients (n = 497) and in the reference group within the total patients and patients with or without concurrent disease

	Total patients			Concurrent disease patients			Previously healthy patients		
	
	ICU patients	Reference group	*P *value	ICU patients	Reference group	*P *value	ICU patients	Reference group	*P *value
Difficulties in falling asleep									
Never or <1 times/week	62%	87%	<0.001	55%	81%	<0.001	78%	94%	<0.001
From 1 to 2 days/week to daily or almost daily	38%	13%		45%	19%		22%	6%	
Total (n)	472	5826		326	2955		146	2871	
Sleep quality during the past month									
Neither good nor bad, good or very good	80%	88%	<0.001	77%	81%	0.080	88%	95%	0.001
Quite bad, poor or very poor	20%	12%		23%	19%		12%	5%	
Total (n)	473	6047		326	3074		147	2973	
Sleep deficit									
Need for sleep higher than habitual sleep	61%	55%	0.034	64%	59%	0.196	56%	51%	0.310
Need for sleep equal or less to habitual sleep	39%	45%		36%	41%		44%	49%	
Total (n)	279	5605		192	2825		88	2780	

Data presented as the percentage of the intensive care unit (ICU) study group (n = 497) (responding at both 6 and 12 months) at the 6-month measure, Not all patients answered the questions.

**Table 4:** Clinical characteristics on admission of all patients with and without sleep disturbances (n = 911)

	Sleep disturbances (n = 419)	No sleep disturbances (n = 471)	*P *value
Age (years)^a^	55.7 (18.4)	60.2 (18.0)	0.419
Gender (male/female) (%)	44.9/50.0	55.1/50.0	0.077
APACHE II score^b^	15.2 (14.4 to 16.0)	15.7 (15.0 to 16.4)	0.525
Duration of stay in ICU (hours)^c^	122.7 (55.0)	126.0 (60.3)	0.878
Duration of stay in hospital (days)^c^	15.2 (9.0)	15.2 (9.0)	0.739
Diagnosis on admission^d^			0.067
Multiple trauma	49 (11.7)	51 (10.8)	
Sepsis	38 (9.1)	38 (8.1)	
Gastrointestinal	80 (19.1)	101 (21.4)	
Respiratory	84 (20.0)	98 (20.8)	
Cardiovascular	29 (6.9)	57 (12.1)	
Miscellaneous	139 (33.2)	126 (26.8)	

Data are given as ^a^mean (standard deviation), ^b^mean (95% confidence interval), ^c^mean and median (standard deviation) or ^d^n (%). APACHE II, Acute Physiology and Chronic Health Evaluation.

### Risk factors for sleep disturbances

Our main findings were that the study group was more likely to have disturbed sleep at both 6 and 12 months (odds ratio = 3.61, 95% confidence interval = 2.93 to 4.46 at 6 months; and odds ratio = 3.62, 95% confidence interval = 2.93 to 4.47 at 12 months for difficulties in falling asleep), and that women had a tendency to have more disturbed sleep at both 6 and 12 months than men (odds ratio = 1.13, 95% confidence interval = 0.98 to 1.30 at 6 months; and odds ratio = 1.16, 95% confidence interval = 1.00 to 1.34 at 12 months for difficulties in falling asleep). Concurrent diseases were strongly associated with all three types of sleep disturbances (odds ratio = 3.34, 95% confidence interval = 2.84 to 3.94 at 6 months; and odds ratio = 3.29, 95% confidence interval = 2.80 to 3.88 at 12 months for difficulties in falling asleep).

### Impact of different factors on sleep disturbances

Concurrent disease was strongly associated with two complaints of sleep disturbances (difficulties in falling asleep and poor quality of sleep) (*P *< 0.001) (Table 5). For the ICU-related factors (APACHE II, length of stay in ICU or in hospital, and admission diagnoses), there were no associations with any of the sleep disturbances. Mechanical ventilation had no significant influence on sleep disturbances (data not shown) (*P *= 0.779 for difficulties in falling asleep, *P *= 0.801 for poor quality of sleep, *P *= 0.512 for sleep deficit).

**Table 5:** Impact of different factors on sleep disturbances at 6 months (n = 911)

		Difficulties in falling asleep			Poor quality of sleep			Sleep deficit		
		
	n	OR	CI 95% for OR	*P *value	OR	CI 95% for OR	*P *value	OR	CI 95% for OR	*P *value
Concurrent disease		2.32	1.67 to 3.23	**<0. 001**	2.51	1.62 to 3.89	**<0. 001**	1.14	0.76 to 1.72	0.52
APACHE II score										
0 to 15	491	1.00		0.06	1.00		0.80	1.00		0.63
16 to 25	315	0.61	0.39 to 0.95		0.85	0.49 to 1.44		1.35	0.71 to 2.55	
26 to 43	105	0.78	0.49 to 1.23		0.83	0.48 to 1.45		1.21	0.63 to 2.32	
LoS in ICU										
<37 hours	221	1.00		0.14	1.00		0.70	1.00		0.33
38 to 52 hours	201	0.98	0.66 to 1.45		1.19	0.74 to 1.91		1.39	0.80 to 2.40	
53 to 144 hours	275	0.64	0.43 to 0.97		0.89	0.54 to 1.47		0.92	0.53 to 1.59	
>144 hours	214	0.85	0.58 to 1.23		0.99	0.63 to 1.57		0.90	0.54 to 1.50	
LoS in hospital										
<5 days	281	1.00		0.75	1.00		0.99	1.00		0.57
6 to 13 days	320	1.00	0.71 to 1.41		0.97	0.64 to 1.46		1.03	0.65 to 1.62	
>13 days	310	0.90	0.64 to 1.25		0.99	0.66 to 1.47		1.25	0.80 to 1.95	
Diagnosis at admission										
Multiple trauma	102	1.00		0.59	1.00		0.14	1.59		0.17
Sepsis	78	1.61	0.85 to 3.03		2.13	0.93 to 4.87		0.98	0.44 to 2.20	
Gastrointestinal	188	1.33	0.69 to 2.56		2.62	1.13 to 6.05		1.99	0.75 to 5.27	
Respiratory	185	1.19	0.69 to 2.08		1.31	0.60 to 2.85		1.35	0.65 to 2.81	
Cardiovascular	88	1.42	0.84 to 2.40		1.97	0.95 to 4.10		1.83	0.91 to 3.69	
Miscellaneous	270	1.10	0.63 to 1.92		1.59	0.74 to 3.40		1.89	0.90 to 3.97	

Impact determined using logistic regression univariate analysis. Intensive care unit (ICU) patients only. The results are adjusted for age and sex. APACHE, Acute Physiology and Chronic Health Evaluation; CI, confidence interval; LoS, length of stay; OR, odds ratio. Bold data is on significant level.

### Health-related quality of life

**Figure 2:**
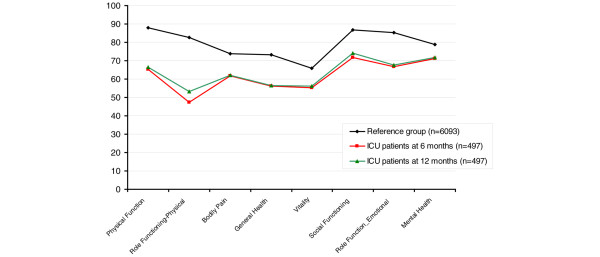
Medical Outcomes Study 36-item Short-form Health Survey results. The Medical Outcomes Study 36-item Short-form Health Survey results are presented for the reference group compared with the intensive care unit (ICU) group that participated at 6 and 12 months. Data presented as the mean.

Baseline SF-36 data for the ICU group are provided in Figure 2. The only correlation in all three aspects of sleep disturbances was found for mental health and bodily pain. Difficulty in falling asleep had an impact on general health. Poor quality of sleep affected vitality. Sleep deficit had an impact on role limitations due to physical problems (Table 6). Increasing age was a risk factor for decreased HRQoL (data not shown).

**Table 6:** Association between sleep disturbances and health-related quality of life at 6 months (n = 911)

	Difficulties in falling asleep			Poor quality of sleep			Sleep deficit		
	
Predictor	OR	95% CI for OR	*P *value	OR	95% CI for OR	*P *value	OR	95% CI for OR	*P *value
Physical functioning	1.0	0.93 to 1.06	0.887	1.08	0.99 to 1.16	0.076	1.04	0.96 to 1.13	0.322
Role limitations due to physical problems	1.01	0.96 to 1.06	0.700	1.04	0.98 to 1.10	0.188	1.08	1.02 to 1.14	0.011
Bodily pain	0.90	0.85 to 0.96	0.001	0.89	0.83 to 0.96	0.002	0.88	0.81 to 0.95	0.001
General health	0.84	0.77 to 0.92	<0.001	0.90	0.80 to 1.02	0.089	0.93	0.83 to 1.04	0.212
Vitality	0.95	0.86 to 1.05	0.318	0.81	0.72 to 0.91	<0.001	0.99	0.88 to 1.12	0.873
Social functioning	1.02	0.95 to 1.10	0.598	1.00	0.91 to 1.10	0.993	1.02	0.92 to 1.12	0.759
Role limitations due to emotional problems	0.98	0.94 to 1.02	0.364	0.95	0.90 to 1.00	0.056	0.99	0.93 to 1.05	0.660
Mental health	0.80	0.74 to 0.87	<0.001	0.80	0.72 to 0.89	<0.001	0.89	0.81 to 0.98	<0.001

Association determined using multiple logistic regression analysis, final model. Adjusted for age and gender. Odds ratio (OR) with a 10-unit change. Intensive care unit patients only. CI, confidence interval.

## Discussion

Our overall aim was to examine the prevalence of long-term sleep disturbances – interpreted as difficulties in falling asleep, poor quality of sleep and sleep deficit – for ICU patients 6 and 12 months after their discharge from the ICU and from the hospital. For the study we used large patient numbers for both the study group and the reference group. The new and important findings are that sleep disturbances are common (up to 38% affected and without improvement at 12 months) after discharge from the ICU and from the hospital. The change in the quality of sleep pattern, however, for the hospitalised patients with an ICU stay was found minor both when comparing patterns prior to ICU stay with after ICU stay as well as patterns 6 months after ICU stay with 12 months after ICU stay. Concurrent disease is the most important factor for sleep disturbances.

### Sleep disturbances

There are few generally accepted definitions or corresponding reference data about sleep disturbances, so the criteria and the reference group must be chosen carefully. We chose the Swedish version of the Basic Nordic Sleep Questionnaire as it has been shown to be practical and valid [15,16] and had also been used to collect the data of the reference group [14]. Our reference group was a large patient group from the referral area of the three hospitals that had also reported similar conditions to those collected for the ICU patient group [4].

Importantly, it was found that few of the patients changed their quality of sleep pattern when comparing patterns prior to the stay with those after the ICU stay and the hospital stay. Also interesting is that one-half of the group that changed their sleep quality showed an improvement. These data suggest that there seem to be only minor changes in sleep quality after a critical care period.

Difficulties in falling asleep and the quality of sleep were affected and remained altered at 12 months in 38% and 20% of former ICU patients, respectively. After adjusting for age and sex, however, it was found that concurrent disease had more effect on the sleep patterns than any other factor.

Like those in the study group, women in the reference group reported more sleep disturbances than men (19% and 16%, respectively) [14]. The predisposition of women for sleep disturbances and the extent of sleep disturbances reported in a Swedish population have previously been confirmed by Fahlen and colleagues [25]. Their study of a general population showed that 23% of the women were affected compared with 14% of men.

It is evident that the long-term sleep disturbances in general for the ICU group are minor at 6 and 12 months, if concurrent disease is excluded from the analysis. When we subtracted the patients who had concurrent disease, we found that there was a 50% reduction in sleep disturbances for the remaining study group. Patients in the ICU are likely to have serious concurrent diseases [4]. Our prestudy hypothesis was that patients in the ICU have more sleep disturbances caused by both the period of critical care and the presence of concurrent diseases. Chronic diseases are known to affect sleeping patterns, and the prevalence of sleep disturbances in such a group in the general population is high [26]. We also found this in the present study, where the overall and most important cause of sleep disturbances was concurrent disease.

We found no relation when we assessed the possible effect of the period of ICU care (APACHE score, length of stay, admission diagnosis, and time on the ventilator) on the sleeping patterns after critical illness. This is in line with the findings of Freedman and colleagues, who found no significant correlations of perceived ICU sleep disturbances and length of stay in ventilated patients or nonventilated patients [17]. Our main finding is therefore that sleeping patterns are altered 6 and 12 months afterwards for people who have been in the ICU, but this is most probably the result of the presence of other diseases rather than of factors related to the care in the ICU itself.

The lack of improvement over time further reduces the likelihood that the period in the ICU contributed appreciably to any sleep disturbances after discharge.

### Health-related quality of life

For the study group we found significantly reduced HRQoL in the dimensions of role limitations due to physical problems, bodily pain, general health, vitality, and mental health measured by the SF-36. These changes correlated only in some aspects to the sleep disturbances.

Comparing our results with other studies is difficult, as we found only one study that had been designed to assess the impact of sleep disturbances on HRQoL after intensive care. In that study, Granja and colleagues used the EuroQol 5D as a measure of HRQoL 6 months after an intensive care stay [27]. They found that sleep disturbances were significantly associated with a worse HRQoL in all dimensions of the EuroQol 5D. Granja and colleagues did not adjust for concurrent disease but 59% of their patients had chronic diseases, and 41% of these reported sleep problems.

Katz and McHorney also assessed the prevalence of insomnia and its impact on HRQoL in patients with chronic illness [12]. They defined insomnia as difficulty in initiating or maintaining sleep; they also showed a close relation between insomnia and chronic illness. Patients with insomnia were independently associated with worsened HRQoL, particularly with worsened mental health, vitality, and general health.

We found that all three types of sleep disturbances affected mental health and bodily pain. Léger and colleagues also found an association between insomnia and bodily pain in their study of HRQoL and insomnia in a general population [28]. They concluded, however, that it is possible for poor sleep to increase the sensitivity to pain. In another study, Schubert and colleagues found that insomnia was common among older adults and that it was then associated with decreased HRQoL [29].

### Limitations of the study

One limitation of the present study is that, in order to evaluate the extent of sleep disturbances in the patient population, we have chosen a control group among inhabitants of the uptake areas of the three hospitals. It may be suggested that a hospitalised group would be a better control group by better picturing the comorbidities. Knowing the heterogeneity of the ICU population, it is very difficult to pick an adjusted cohort containing the specific characteristics of our ICU population, especially as large numbers are needed. We have chosen a more practical solution – that is, to address a very large number of habitants in the area. In order to adjust the individuals in this cohort to concurrent disease, they were asked to provide information on factors believed to be important for their health. The individuals have provided diagnoses and symptoms; the latter was converted to diagnoses by two medicine doctors [4]. We have thereafter tried to make a comparison between the patients and this adjusted cohort. As this group is only an attempt to compensate for not having the sleep disturbances data prior to the ICU stay, it is a shortcoming of the present study.

Secondly, the ICU length of stay is short in the present study. Although the length of stay is comparable with the length of stay presented in the Swedish ICU registry, it may be significantly shorter than seen for other ICUs. This precludes its generalisability for such settings.

There is limited information on the reliability of and validity of sleep questionnaires in the critical care setting. There is also no consensus on which protocol to use. Further, there is the risk of recall bias – although this bias can be argued to be minor as there are 6 months between the measurements. These three listed factors may also hamper the evaluation of the data.

Fourthly, it is important for the strengths of the conclusions made in the present paper to note that there is a significant loss to follow-up. The low response rate, however, is in the range commonly seen in similar studies.

Fifth, an important influence on sleep disturbances is the degree of substance use or misuse [30]. We did not ask the patients specifically if they misused alcohol or other substances or drugs. This may have influenced our findings if substance misuse had been higher in the ICU group than in the reference group, as such effects may lead to a misleadingly high rate of sleep disturbances [30]. As we were unable to find any effects beyond those of age, sex, and concurrent disease, however, such factors may be claimed less important. Furthermore, the extent of sedation during the ICU period may also be claimed as an important factor for our outcome. Using the time on ventilator as a surrogate measure of the extent of sedation, however, we were unable to find any correlations to sleep disturbances.

Another limitation in our study is that we did not assess post-traumatic stress disorder. Complaints of sleep disturbances are common among patients with post-traumatic stress disorder, and the disorder is common in patients who have been treated in the ICU [31]. As the effects beyond the factors examined and adjusted for (age, sex, concurrent disease and ICU-related factors) were minor, however, we think the overall effect of post-traumatic stress disorder must also be limited. In addition, Klein and colleagues demonstrated in their study of motor-vehicle-collision victims that altered perception rather than sleep disturbance *per se *may be the key problem in post-traumatic stress disorder [7].

Finally, effects of cognitive function or dysfunction may have affected the results and their interpretation. Unfortunately, the present study did not assess this.

## Conclusion

Although the change in quality of sleep prior to the ICU and hospital stays compared with that after the ICU and hospital stays seem to be minor, we found a high prevalence of sleep disturbances (difficulties in falling asleep, quality of sleep and sleep deficit) for the patient long term after discharge from the ICU. Interestingly, these sleep disturbances were not affected by ICU factors but were instead mostly due to concurrent diseases. It is thus important to include assessment of concurrent diseases in sleep-related research for the ICU population.

## Key messages

• Changes in quality of sleep prior to compared with after stays in the ICU and in the hospital seem to be minor.

• Sleep disturbances are common after critical care at 6 months (from 5% to 25% more common than the general population), with little or no improvement over time.

• Intensive-care-related factors do not seem to influence sleep at 6 and 12 months after ICU stay, whereas concurrent disease is the main explanation for the sleep problems registered.

## Abbreviations

APACHE II = Acute Physiology and Chronic Health Evaluation; HRQoL = health-related quality of life; ICU = intensive care unit; SF-36 = Medical Outcomes Study 36-item Short-form Health Survey.

## Competing interests

The authors declare that they have no competing interests.

## Authors' contributions

LO designed the study, performed and interpreted the data analysis, and drafted the manuscript. AN and FS designed the study, interpreted the data analysis, and drafted the manuscript. PN and UE-G revised the manuscript. All authors have read and approved the final manuscript.

## Supplementary Material

Additional file 1 An Excel file presenting the sleep instruments used in the present study. Click here for file
